# Validation of an enzyme-linked immunosorbent assay for the quantification of citrullinated histone H3 as a marker for neutrophil extracellular traps in human plasma

**DOI:** 10.1007/s12026-017-8905-3

**Published:** 2017-02-04

**Authors:** Charlotte Thålin, Maud Daleskog, Sophie Paues Göransson, Daphne Schatzberg, Julie Lasselin, Ann-Charlotte Laska, Anders Kallner, Thomas Helleday, Håkan Wallén, Mélanie Demers

**Affiliations:** 1 Department of Clinical Sciences, Danderyd Hospital, Division of Internal Medicine, Karolinska Institutet, Stockholm, Sweden; 2 Department of Clinical Sciences, Danderyd Hospital, Department of Anesthesia and Intensive Care, Karolinska Institutet, Stockholm, Sweden; 3 Department of Biology, Boston University, Boston, MA 02215 USA; 4 Stress Research Institute, Stockholm University, Stockholm, Sweden; 5 Department of Clinical Neuroscience, Division of Psychology, Karolinska Institutet, Solna, Stockholm, Sweden; 6 Department of Clinical Chemistry, Karolinska University Hospital, Stockholm, Sweden; 7 Department of Medical Biochemistry and Biophysics, Division of Translational Medicine and Chemical Biology, Karolinska Institutet, Science for Life Laboratory, Stockholm, Sweden; 8 Department of Clinical Sciences, Danderyd Hospital, Division of Cardiovascular Medicine, Karolinska Institutet, Stockholm, Sweden

**Keywords:** PAD4, H3Cit, NETs, Elisa, Human plasma, LPS-induced inflammation

## Abstract

There is an emerging interest in the diverse functions of neutrophil extracellular traps (NETs) in a variety of disease settings. However, data on circulating NETs rely largely upon surrogate NET markers such as cell-free DNA, nucleosomes, and NET-associated enzymes. Citrullination of histone H3 by peptidyl arginine deiminase 4 (PAD4) is central for NET formation, and citrullinated histone H3 (H3Cit) is considered a NET-specific biomarker. We therefore aimed to optimize and validate a new enzyme-linked immunosorbent assay (ELISA) to quantify the levels of H3Cit in human plasma. A standard curve made of in vitro PAD4-citrullinated histones H3 allows for the quantification of H3Cit in plasma using an anti-histone antibody as capture antibody and an anti-histone H3 citrulline antibody for detection. The assay was evaluated for linearity, stability, specificity, and precision on plasma samples obtained from a human model of inflammation before and after lipopolysaccharide injection. The results revealed linearity and high specificity demonstrated by the inability of detecting non-citrullinated histone H3. Coefficients of variation for intra- and inter-assay variability ranged from 2.1 to 5.1% and from 5.8 to 13.5%, respectively, allowing for a high precision. Furthermore, our results support an inflammatory induction of a systemic NET burden by showing, for the first time, clear intra-individual elevations of plasma H3Cit in a human model of lipopolysaccharide-induced inflammation. Taken together, our work demonstrates the development of a new method for the quantification of H3Cit by ELISA that can reliably be used for the detection of NETs in human plasma.

## Introduction

Neutrophil extracellular traps (NETs) are webs of chromatin fibers (DNA and histones) coated with antimicrobial granular proteins including the enzymes neutrophil elastase (NE) and myeloperoxidase (MPO). Released by neutrophils into the extracellular space upon activation, NETs were discovered to trap and kill bacteria as part of the innate immune system over a decade ago [1] and have since then been implicated in several pathological conditions. In addition to a pro-thrombotic activity in deep vein thrombosis [2, 3], acute coronary syndrome [4–6] and ischemic stroke [7–9], NETs have been shown to impair fibrinolysis and induce tissue and organ damage in sepsis [10, 11], promote the autoimmune response in small vessel vasculitis [12], contribute to endothelial damage in systemic lupus erythematosus [13, 14], and acute lung injury [15], as well as impair wound healing in diabetes [16]. A role in cancer is also emerging, where NETs have been implicated in cancer-associated thrombosis [17], tumor growth, and progression [18, 19]. In light of the emerging data on the adverse role of NETs, pre-clinical studies are now starting to explore the possibility of alleviating the effects of NETs with new therapeutic agents that degrade NETs or inhibit their formation [3, 8, 20]. In this context, a reliable and specific biomarker of NETs would play a central role in prediction of risk, prognosis, and therapeutic effects.

Studies of NET formation in the above disease settings rely largely upon in vitro stimulation of neutrophils and subsequent NET formation assessing the susceptibility of neutrophils to undergo NETosis. Quantification of surrogate NET markers in plasma, such as cell-free DNA (cfDNA), nucleosomes, and the NET-associated enzymes NE and MPO by commercially available enzyme-linked immunosorbent assay (ELISA) kits, has also been implemented. Data obtained with these assays should be interpreted with caution, as events unrelated to NETosis, such as tissue injury, apoptosis, and necrosis, may generate circulating cfDNA as well as nucleosomes, whereas circulating NE and MPO may reflect neutrophil and/or macrophage activation not related to NET generation. Some studies also identified circulating levels of MPO-DNA complexes using a capture ELISA [11, 12, 21]. However, MPO is a highly positively charged secreted protein [22], which can bind to the negatively charged cfDNA released in the plasma following tissue injury, thus questioning its specificity as a NET marker.

Prior to releasing NETs, peptidylarginine deiminase 4 (PAD4), an enzyme that is primarily expressed in neutrophils, translocates to the nucleus and converts peptidylarginine to peptidylcitrulline on histone H3. The citrullination of positively charged arginine residues leads to uncharged citrulline residues, loss of ionic interactions, and subsequent chromatin decondensation, the initial step of NETosis. Citrullinated Histone H3 (H3Cit) is thereby considered a NET-specific biomarker [23].

An assay to estimate the levels of the NET biomarker H3Cit in plasma would allow for a more specific assessment of a circulating NET burden. We therefore aimed to validate and optimize an ELISA-based assay recently shown to detect H3Cit in plasma of patients with ischemic stroke [9].

## Materials and methods

### Reagents and equipment

Microplates with 96 streptavidin pre-coated wells, monoclonal anti-histone-biotin antibodies, and incubation buffer (all from Cell Death Detection ELISA PLUS kit, Roche, Cat. No. 11 774 425 001). Phosphate buffered saline (PBS; Life Technologies, Cat. No. 14190-250), tween 20 (Sigma-Aldrich, Cat. No. A9418), rabbit polyclonal anti-histone H3 (citrulline R2 + R8 + R17) antibody (Abcam, Cat. No. AB5103), bovine serum albumin, BSA (Sigma-Aldrich, Cat. No. A9418), goat anti-rabbit IgG horseradish-peroxidase (HRP) conjugate (BioRad, Cat. No. 170-6515), 3,3′, 5,5′-tetramethylbenzidine (TMB) liquid substrate (Sigma-Aldrich, Cat. No. T0440), stop solution (Thermo Scientific, Cat. No. N600), Trizma base (Sigma-Aldrich, Cat. No. T1503), CaCl_2_ (Sigma-Aldrich C1016), phenylmethylsulfonyl fluoride (PMSF) protease inhibitor (Life Technologies, Cat. No. 36978), dithiothreitol, DTT (Invitrogen, Cat. No. P2325), human recombinant PAD4 (Cayman Chemical, Cat. No. 10500), human recombinant histone H3 (Cayman Chemical, Cat. No. 10263), ELISA reader (Tecan Sunrise)

### Preparation of standard

A working stock solution of H3Cit was made as described previously [24]. Briefly, human recombinant PAD4 and human recombinant histones H3 at a ratio 2.5 U of PAD4 per microgram of histones were incubated at 37 °C for 1 h in reaction buffer (50 mM Trizma base with 4 mM CaCl_2_, pH 7.6, 4 mM DTT, and 1 mM PMSF). A final concentration of 10,000 ng/mL H3Cit was obtained by adding PBS-1% BSA. The stock solution was aliquoted, frozen on dry ice, and stored at −80 °C until later use.

### Samples

Samples were taken from healthy individuals prior to and 3–4 h after receiving intravenous injection of lipopolysaccharide (LPS; 2 ng/kg of body weight *Escherichia coli* endotoxin, Lot H0K354 CAT number 1235503, United States Pharmacopeia, Rockville, MD, USA) or from healthy volunteers. Plasma samples were prepared from citrated whole blood following immediate centrifugation for 20 min at 2000×*g* after which they were stored at −80 °C until further analysis. At time of anal-ysis, samples were thawed on ice and diluted 1:2 in PBS unless otherwise indicated. All study individuals gave written informed consent for the use of their plasma, and the study complied with the Declaration of Helsinki.

### ELISA methodology

The microplate and diluents were kept at room temperature 30 min prior to starting the assay. Stock solution, antibodies, and samples were thawed on ice and kept on ice until loading of microplate. All incubations were at room temperature and washes were repeated four times with PBS-Tween (0.05%) with 20 s soaking for each wash. The concentrations of the standard curve, incubation times, and dilutions of samples were optimized in preliminary experiments.

**Fig. 1:**
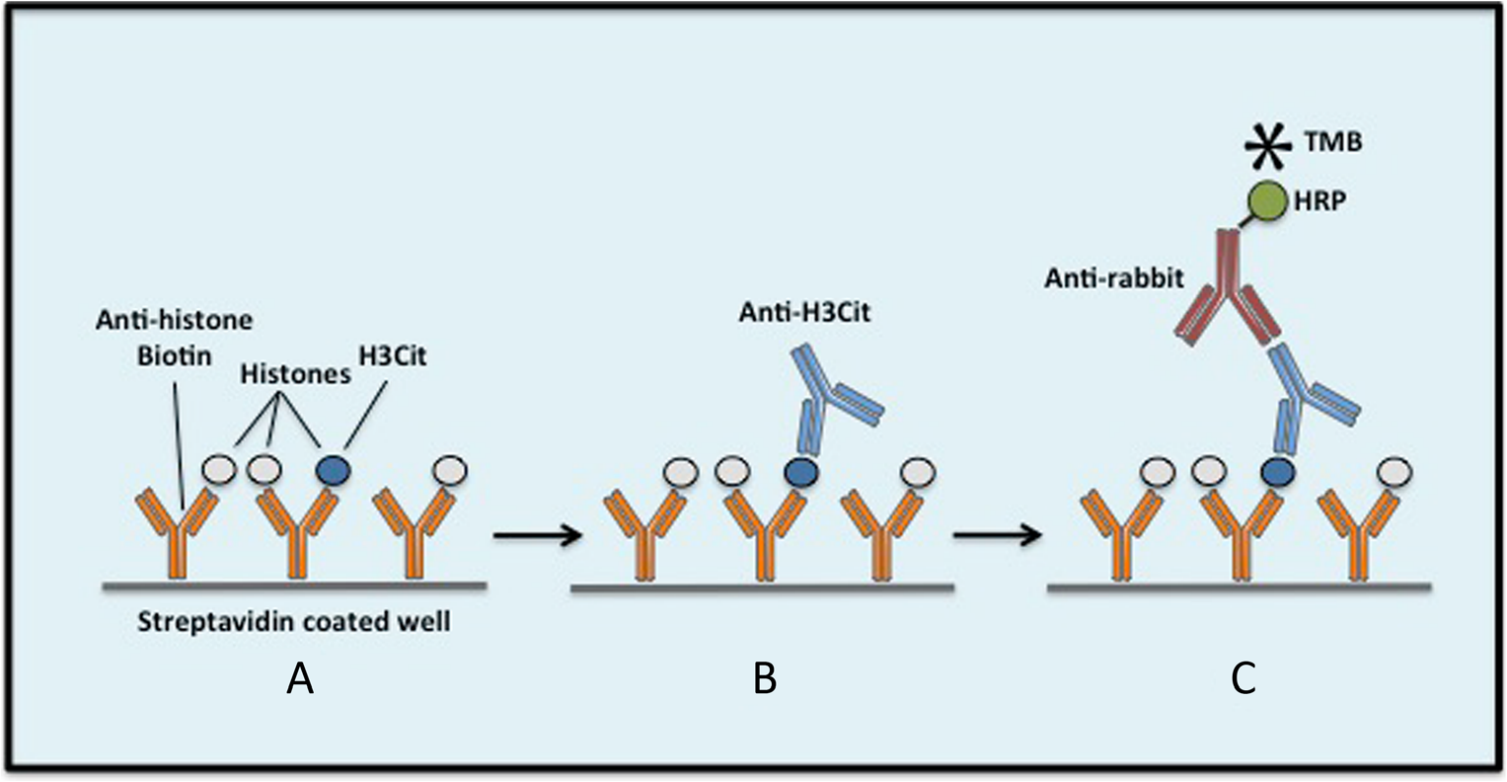
Schematic of the H3Cit ELISA procedure. *A* Anti-histone biotin (the capture antibody) is coated to streptavidin pre-coated wells during the first incubation. Samples are pipetted into the wells and histones bind to the capture antibody during the second incubation. *B* After washing, anti-H3Cit is added to the wells, binding to immobilized H3Cit but not to histones H3 that are not citrullinated, during the third incubation. *C* In the fourth incubation, an HRP conjugated anti-rabbit antibody is added and binds to the anti-H3Cit, after which TMB is added for detection

The assay was performed as follows (Fig. 1): 100 μL of anti-histone biotin (1:10 in incubation buffer) was added to Streptavidin pre-coated wells and incubated for 2 h. After washing, 50 μL of standard solutions or samples was added to each well and incubated for 1.5 h, then washed again. 100 μL of anti-histone H3 (citrulline R2 + R8 + R17; anti-H3Cit) antibody (1:2000 in 1% BSA in PBS) was applied to each well for 1 h incubation. After washing, the wells were incubated for another hour with 100 μL anti-rabbit HRP conjugate antibody (1:5000 in 1% BSA in PBS), followed by washing. For detection, 100 μL TMB was added to each well and incubated for 20 min in the dark. The reaction was stopped by adding 50 μL stop solution. The optical density (O.D.) was measured at a wavelength of 450 nm with a reference correction wavelength at 620 nm using an automatic plate reader.

### Assay validation

For validation of the assay, we assessed the following: linearity, stability, limit of detection, specificity, recovery, and precision. Trueness could not be determined as no reference analyte of known concentration is available, and there is no available assay for the quantification of H3Cit in plasma for comparison. The linear interval was defined as the linear section of the best-fit standard curve. Each standard curve was fitted using a four-parameter logistic (4PL) regression, and the 95% confidence interval (95% CI) was considered. The limit of detection was approximated from the intersection of the lower asymptote of the upper 95% CI with the 4PL fit of the standard data. Specificity was assessed by the ability to detect citrullinated histone H3 but not non-citrullinated histone H3 in similar conditions by preparing a standard without PAD4, thus preventing the citrullination of histone H3. Recovery and the effect of the matrix were assessed by spiking plasma samples from four healthy volunteers with known concentrations of in vitro PAD4-citrullinated histone H3, comparing this to the detector response obtained for the same concentrations of in vitro PAD4-citrullinated histone H3 diluted in PBS-1% BSA. Precision was expressed by the intra- and inter-assay coefficient of variation (%CV, defined as the ratio between standard deviation and mean value). The maximum accepted %CV for intra- and inter-assay variability were set to 15%. Stability was assessed by comparing the detector response obtained from freshly prepared and frozen aliquots of H3Cit standard and comparing standard curves from frozen aliquots from three different batches of H3Cit that had been citrullinated on three different days. One versus two freeze-thaw cycles of plasma samples were also compared.

### Statistical analyses

O.D. was fitted versus nominal log concentration applying a sigmoidal 4PL regression to the calibration curve. 4PL curves were compared by *F*-test. Data were analyzed using GraphPad Prism 6 (GraphPad Software, Inc., La Jolla, CA, USA).

## Results

### Standard preparation and linearity

**Fig. 2:**
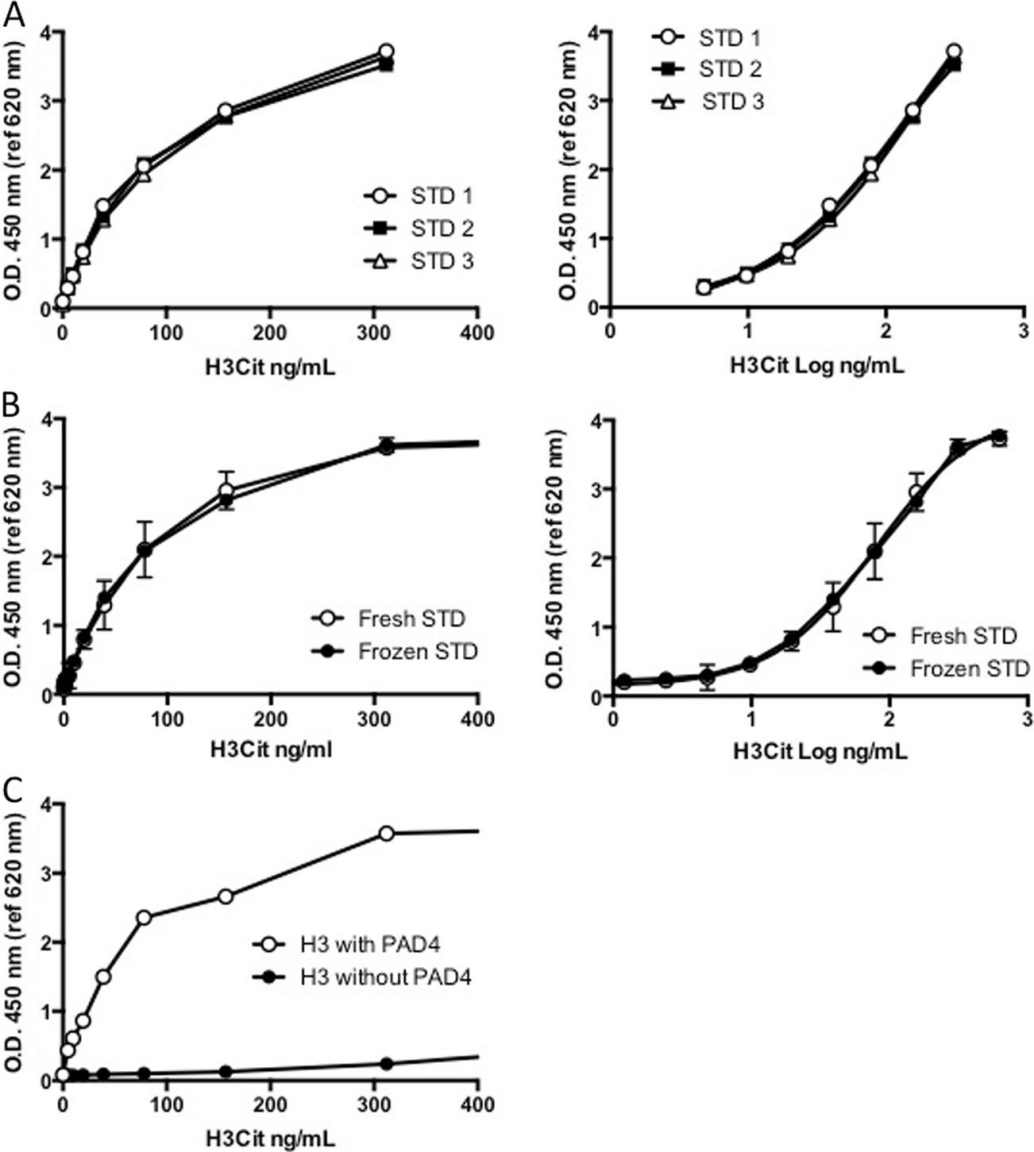
In vitro PAD4-citrullinated histone H3 standard. **a** Standard curves. The detector response when preparing standard curves from frozen aliquots from three different batches of PAD4-citrullinated histone H3 on three different days (STD 1–3) were not significantly different (*F* (DFn, DFd) = 2.6 (8, 9); *p* = 0.088). **b** Standard curves generated from freshly made or frozen aliquot of H3Cit standards. No significant difference was observed when comparing the detector response of the freshly made versus frozen standards (*F* (DFn, DFd) = 0.2 (4, 52); *p* = 0.916. **c** Data obtained when a standard curve was prepared with histone H3 incubated in the same conditions as our standard preparation of H3Cit, but without PAD4, rendering non-citrullinated histones, representative of three different experiments. There was a low amount of antibody antigen detection when large amounts of non-citrullinated histone H3 were present, but the antibody antigen detection was specific for H3Cit in the linear interval of the assay

As no international standard preparation is available for H3Cit, we generated a standard curve using in vitro PAD4-citrullinated H3Cit, as previously described [24]. The stock was serially diluted 1:2 in PBS-1% BSA to obtain a standard curve and applied to a streptavidin-coated plate using an anti-histone biotin antibody as capture and an anti-H3Cit antibody for detection. To determine the suitable linear interval, we interpolated the detected O.D. from the serial dilutions of H3Cit to different regressions. The best-fit curve was a sigmoidal 4PL curve rendering a linear interval of the curve between ≈ 0.3 and 3.5 O.D., corresponding to concentrations between ≈5 and ≈300 ng/mL (Fig. 2a).

### Stability

The detector response when preparing standards from freshly citrullinated H3Cit was very similar to the detector response obtained from frozen aliquots of the same standards (Fig. 2b). Moreover, the detector response when preparing standard curves from frozen aliquots from three different batches of H3Cit citrullinated on three different days were not significantly different (Fig. 2a), allowing for a good reproducibility.

### Limit of detection

To determine the limit of detection, we approximated the lowest detectable concentration determined from the curve to ≈5 ng/mL. This concentration corresponded to the intersection of the lower asymptote of the upper 95% CI with the 4PL fit of the standard curve. The limit of detection with stated probability was therefore set to approximately 5 ng/mL.

### Specificity

To assess the specificity of the assay, we prepared a standard curve with histone H3 incubated under the same conditions as our standard preparation of H3Cit, but without PAD4, rendering non-citrullinated histones, and compared this to our standard curve with in vitro PAD4-citrullinated H3Cit. Although there was a low amount of antibody antigen detection when large amounts of non-citrullinated histone H3 were present, the antibody antigen detection was specific for citrullinated H3Cit in the linear interval of the assay (Fig. 2c).

### Effect of the matrix

**Fig. 3:**
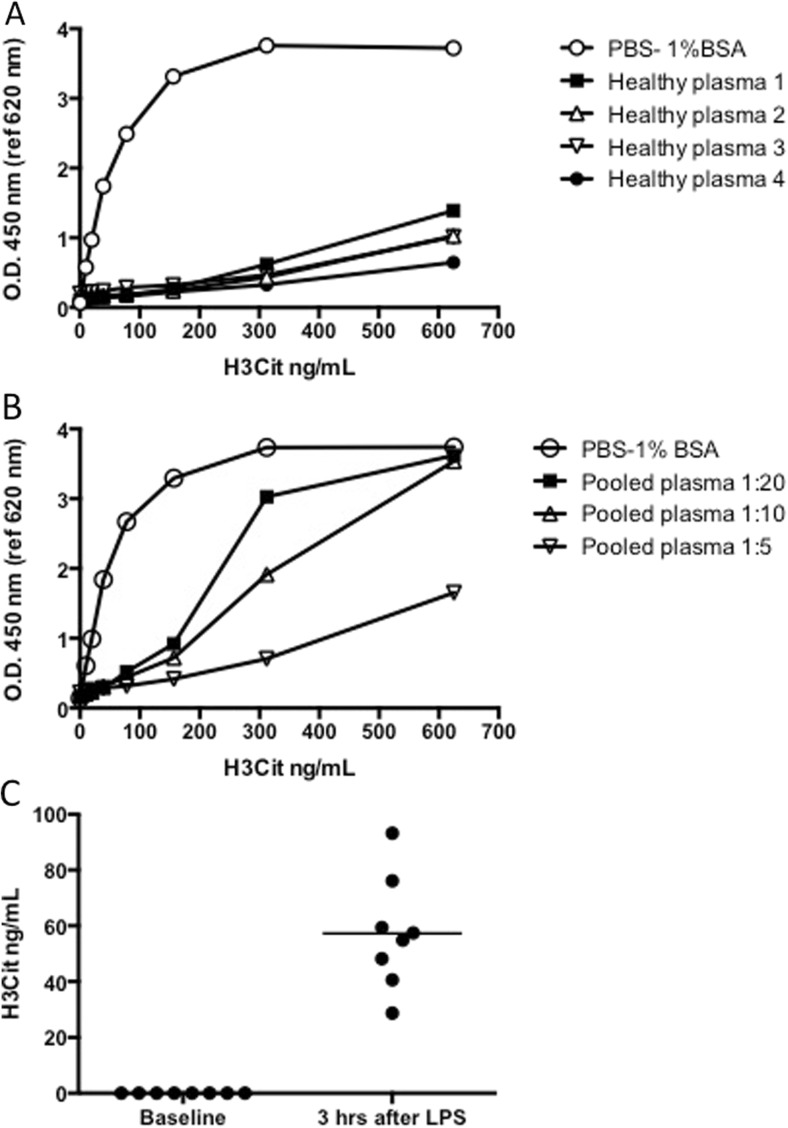
Detection of H3Cit in plasma samples. **a** At baseline, no H3Cit was detected in plasma from healthy volunteers, whereas the spiking of known concentrations of H3Cit into these plasmas diluted 1:2 gave a significantly lower detector response compared to the detector response obtained from the standard diluted in PBS-1% BSA, suggesting an effect of the matrix. **b** Standards prepared from H3Cit diluted in pooled plasma from healthy donors at various dilutions, rendered an obvious increase in detector response with increasing dilutions of plasma. **c** The quantification of H3Cit in plasma of healthy volunteers before LPS injections were under the detection limit of approximately 5 ng/mL. An increase in the levels of H3Cit in all plasma samples taken from the same individuals 3–4 h after LPS was observed, ranging from 28.7 to 93.2 ng/mL

To evaluate whether components of the sample matrix (i.e., plasma), such as proteins, phospholipids, carbohydrates, or various metabolites, interfered with the binding of H3Cit to either the capture antibody or the detection antibody, we spiked known concentrations of H3Cit to plasma diluted 1:2 from four healthy volunteers. This gave a significantly lower detector response compared to the detector response obtained from the standard diluted in PBS-1% BSA (Fig. 3a), suggesting an effect of the matrix. To further study this effect, we prepared the standard in pooled plasma from healthy donors diluted 1:20, 1:10, and 1:5 in PBS, rendering a dose response of the detector with increasing dilutions of plasma (Fig. 3b). However, the citrullinated histones used for these spiking experiments were free citrullinated histones, as opposed to the citrullinated histones in our samples which are hypothesized to be bound to cfDNA in nucleosomes, suggesting that there are components in plasma either interfering with the antibody detection of free histones or degrading free histones in plasma, aggravating the attempt to recover free histones in plasma.

### Concentrations of H3Cit in plasma in a human model of LPS-induced inflammation

Surrogate markers of NETs (cfDNA, nucleosomes and MPO-DNA complexes) have been identified in the plasma of septic patients [10, 11, 25, 26] and in murine models of lipopolysaccharide (LPS)-induced septic shock [11, 27, 28]. Furthermore, H3Cit was detected by western blot in plasma of mice shortly after LPS injection [27, 28]. With the intention to perform the assay validation with samples containing H3Cit, we therefore used samples from healthy volunteers receiving intravenous LPS in an experimental model of inflammation. The samples were taken at baseline (before LPS injection) and after 3–4 h, with the hypothesis that LPS injection would induce a systemic NET formation resulting in elevated and detectable levels of H3Cit in plasma. Indeed, the levels of H3Cit in all samples taken at baseline were under the detection limit of approximately 5 ng/mL, and the levels of H3Cit in all samples taken from the same individuals 3–4 h after LPS injection ranged from 28.7 to 93.2 ng/mL (Fig. 3c). These concentrations were all calculated from detection of optical density within the linear interval of the standard curve following a 1:2 dilution of plasma samples (Fig. 2a). However, repeated freeze-thaw cycles of plasma samples with known concentrations of H3Cit rendered a mean reduction of 13.4 ± 2.3% after a second freeze-thaw cycle. Freeze-thaw cycles of the plasma are therefore not recommended when applying this assay.

### Precision and reproducibility

To assess the precision of the assay, we performed the assay on six replicates of eight samples (1-8) within the same assay run as well as duplicates of the same eight samples in four different assay runs performed on four different days. The CV were all <15%, with the intra-assay ranging from 2.13 to 5.15% and the inter-assay ranging from 5.80 to 12.55%, showing a high precision with good repeatability and reproducibility of the assay (Table 1).

**Table 1:** Precision, intra-assay repeatability, and four different days inter-assay reproducibility

Sample	1	2	3	4	5	6	7	8
Coefficient of variation (%) intra-assay (*n* = 6)	5.1	4.5	5.08	2.7	4.35	3.58	2.13	3.1
Coefficient of variation (%) inter-assay (*n* = 4)	11.54	10.27	12.55	8.5	9.6	10.53	5.8	13.5

## Discussion

Our study establishes an assay allowing for the fast and reliable quantification of the NET-specific biomarker H3Cit in human plasma. We also show, for the first time, an elevation of H3Cit in plasma in a human model of LPS-induced inflammation.

The validation of the assay revealed a high specificity for H3Cit as well as a high stability of the custom-made standard, rendering a good precision and reproducibility. Although we show a clear dose-dependent effect of the matrix on the detection of free citrullinated histones added to plasma, we can only speculate on whether the citrullinated histones in our samples are protected by surrounding DNA as part of nucleosomes. Free histones are highly positively charged and have been shown to bind to negatively charged components such as proteins and heparins in plasma [29], potentially blocking the binding sites of the antibodies in the assay. Free histones have also been shown to bind to phospholipids such as phosphatidylserine and phosphatidylethanolamine present on microparticles [30], as well as to platelets [31] and platelet adhesion molecules such as vWF and fibrinogen [32]. Furthermore, histones could in their free form be subject to rapid degradation by free proteases within the plasma such as activated protein C [33], or the NET-associated enzymes NE, MPO [34], and cathepsins [35]. Indeed, the degradation of free histones in plasma was recently shown by western blotting assessing histone degradation over time in plasma from healthy volunteers spiked with free calf thymus histones, revealing a very rapid degradation with a half-life of 4.6 min [36]. However, H3Cit bound to DNA in nucleosomes, the endpoint of interest in the detection and quantification of circulating NETs, could be protected against degradation and/or further binding and subsequent blocking of the antibody binding site in the assay. It is therefore our hypothesis that the amount of H3Cit quantified by this assay is in fact the amount of H3Cit protected by the NET complex, excluding a possible portion of free H3Cit in plasma. On the other hand, the standard of H3Cit used in our assay comprises in vitro citrullinated H3Cit, and the concentration of H3Cit in the standard curve is therefore an estimation based on the assumption that all histones were citrullinated, assuming the optimal enzymatic activity of PAD4 at a ratio of 2.5 U/μg of histones. A possible underestimation of the concentration in our samples can therefore not be ruled out. However, the assay provides a reproducible estimate of the concentrations of detectable H3Cit in plasma with high specificity, stability, and precision, rendering a robust and reliable assay for the comparison of the levels of detectable H3Cit in human plasma.

Interestingly, all samples taken at baseline in the LPS-induced model of inflammation were below the limit of detection, suggesting that healthy people do not have a baseline systemic NET burden. This is in line with a recent study revealing a low amount of H3Cit in plasma of healthy individuals when applying a similar assay but without concentrations derived from a standard curve [9]. Furthermore, in accordance with previous studies showing elevations of surrogate markers of NETs in septic patients [10, 11, 25, 26] as well as the detection of H3Cit by western blotting in murine models of LPS-induced septic shock [27, 28], our results support an inflammatory induction of a systemic NET burden by showing clear intra-individual elevations of H3Cit in healthy individuals receiving LPS injection. Further studies are now warranted to confirm these elevations in a clinical setting of sepsis.

There is an emerging interest in the role of NETs in various disease settings. Apart from its role as a central player of the innate immune system in both bacterial [1, 10, 11, 25–28, 33, 36] and viral infection [15, 37], NETs are now being implicated in several common and widespread diseases such as arterial and venous thrombosis [2–9], cancer [17–19], and diabetes [16]. Prior studies rely largely upon the detection and quantification of surrogate markers of NETs, such as cfDNA, NE, and MPO. There is therefore an unmet need for a more specific assessment of a systemic NET burden to explore the potential of NETs in disease prediction and progression. Moreover, with the use of a detection antibody widely shown to recognize citrullinated histone H3 in plasma and cells from mouse and human [9, 16, 17, 19, 38] we believe that our method has the potential to detect NET burden not only in human plasma but also in disease mouse models and in vitro research.

In conclusion, we believe that this assay could be of great value in further studies of a systemic NET burden. If quantifiable levels of H3Cit in plasma prove to be useful as a prognostic marker in conditions such as sepsis or prediction of diseases such as thrombosis and cancer, further development of this assay would allow for its implementation in several clinically important settings.
